# Effect of Interleukin-17 in the Activation of Monocyte Subsets in Patients with ST-Segment Elevation Myocardial Infarction

**DOI:** 10.1155/2020/5692829

**Published:** 2020-06-27

**Authors:** Montserrat Guadalupe Garza-Reyes, Mónica Daniela Mora-Ruíz, Luis Chávez-Sánchez, Alejandra Madrid-Miller, Alberto Jose Cabrera-Quintero, José Luis Maravillas-Montero, Alejandro Zentella-Dehesa, Luis Moreno-Ruíz, Selene Pastor-Salgado, Erick Ramírez-Arias, Nataly Pérez-Velázquez, Adriana Karina Chávez-Rueda, Francisco Blanco-Favela, Wendy Guadalupe Vazquez-Gonzalez, Alicia Contreras-Rodríguez

**Affiliations:** ^1^Unidad de Investigación Médica en Inmunología, UMAE, Hospital de Pediatría, Centro Médico Nacional Siglo XXI, Instituto Mexicano del Seguro Social, Ciudad de México, Mexico; ^2^División de Programas Educativos de la Coordinación de Educación en Salud, Instituto Mexicano del Seguro Social, Ciudad de México, Mexico; ^3^Unidad de Bioquímica, Instituto Nacional de Ciencias Médicas y Nutrición Salvador Zubirán and Instituto de Investigaciones Biomédicas, Universidad Nacional Autónoma de México, Programa Institucional de Cáncer de Mama, Depto. Medicina Genómica y Toxicología Ambiental, Instituto de Investigaciones Biomédicas, Universidad Nacional Autónoma de México, Ciudad de México, Mexico; ^4^Red de Apoyo a la Investigación, Universidad Nacional Autónoma de México and Instituto Nacional de Ciencias Médicas y Nutrición, Salvador Zubirán, Ciudad de México, Mexico; ^5^Servicio de Cardiología Adultos, División de Cardiología de la Unidad Médica de Alta Especialidad, Hospital de Cardiología, Centro Médico Nacional Siglo XXI, Instituto Mexicano del Seguro Social, Ciudad de México, Mexico; ^6^Servicio de Urgencias de la Unidad Médica de Alta Especialidad, Hospital de Cardiología, Centro Médico Nacional Siglo XXI, Instituto Mexicano del Seguro Social, Ciudad de México, Mexico; ^7^Servicio de Cardiología de la Unidad Médica de Alta Especialidad, Hospital de Especialidades, Dr. Ignacio García Téllez, Instituto Mexicano del Seguro Social, Mérida, Yucatán, Mexico; ^8^Servicio de Gabinetes de la Unidad Médica de Alta Especialidad, Hospital de Cardiología, Centro Médico Nacional Siglo XXI, Instituto Mexicano del Seguro Social, Ciudad de México, Mexico

## Abstract

Interleukin- (IL-) 17 is increased in acute myocardial infarction (AMI) and plays a key role in inflammatory diseases through its involvement in the activation of leukocytes. Here, we describe for the first time the effect of IL-17 in the migration and activation of monocyte subsets in patients during ST-segment elevation myocardial infarction (STEMI) and post-STEMI. We analyzed the circulating levels of IL-17 in patient plasma. A gradual increase in IL-17 was found in STEMI and post-STEMI patients. Additionally, IL-17 had a powerful effect on the recruitment of CD14^++^CD16^+^/CD14^+^CD16^++^ monocytes derived from patients post-STEMI compared with the monocytes from patients with STEMI, suggesting that IL-17 recruits monocytes with inflammatory activity post-STEMI. Furthermore, IL-17 increased the expression of TLR4 on CD14*^+^*CD16*^−^* and CD14^++^CD16^+^/CD14^+^CD16^++^ monocytes post-STEMI and might enhance the response to danger-associated molecular patterns post-STEMI. Moreover, IL-17 induced secretion of IL-6 from CD14^++^CD16^−^ and CD14^++^CD16^+^/CD14^+^CD16^++^ monocytes both in STEMI and in post-STEMI, which indicates that IL-17 has an effect on the secretion of proinflammatory cytokines from monocytes during STEMI and post-STEMI. Overall, we demonstrate that in STEMI and post-STEMI, IL-17 is increased and induces the migration and activation of monocyte subsets, possibly contributing to the inflammatory response through TLR4 and IL-6 secretion.

## 1. Introduction

Acute coronary syndromes comprise the acute manifestations of coronary artery disease, including ST-segment elevation myocardial infarction (STEMI), which in the majority of cases occurs from a complete thrombotic occlusion developing from an atherosclerotic plaque in an epicardial coronary vessel and is associated with great morbidity and mortality [1]. In the first days of STEMI, a strong inflammatory response is induced that involves an increased release of several cytokines and infiltration of leukocytes in the heart tissue [2], followed by a second phase starting on day 4 (post-STEMI) that is maintained for several days[3] .

After myocardial infarction, the monocyte subset (CD14^++^CD16^−^, CD14^++^CD16^+^, and CD14^+^CD16^++^) numbers in the circulation increase in patients with STEMI [4]. These cells release inflammatory mediators, such as tumor necrosis factor- (TNF-) *α*, interleukin- (IL-) 1, and IL-6, which contribute to myocardial injury [2]. Furthermore, previous studies have shown that CD14^++^CD16^−^ monocytes predict cardiovascular events [5] and that CD14^+^CD16^−^ monocytes predominate in the infarct border zone [6]. In addition, human monocytes express patterns of genes associated with inflammation after acute myocardial infarction (AMI) [7], suggesting that these populations of monocytes have a relevant role in AMI in humans.

Inflammatory cytokines in AMI potently activate endothelial cells, increase expression of adhesion molecules on endothelial cells, and promote the activation of leukocytes to enhance the inflammatory response. IL-17 is an inflammatory cytokine produced by a variety of cell types, including macrophages and T cells [8, 9]. IL-17 contributes to the expression of molecules such as IL-8 and CCL2 and increases that of intercellular adhesion molecule- (ICAM-) 1 on endothelial cells [8, 9]. It has also been proposed that IL-17 contributes to the recruitment of neutrophils and monocytes [10, 11] and even induces the activation of human macrophages, which secrete cytokines such as IL-1*β* and TNF-*α* [12]. IL-17 levels are increased in the plasma and tissues such as the aorta of apolipoprotein E-deficient (Apoe^−/−^) mice, promoting monocyte recruitment into lesions, and blockade of the effect of IL-17A in Apoe^−/−^ mice reduces atherosclerotic plaque burden. In humans, higher levels of IL-17 have been found in patients with AMI than in those with unstable angina or stable angina [13, 14].

The dynamics of monocyte subsets and levels of IL-17 post-STEMI have been reported. However, the role of IL-17 in the activation of monocyte subsets derived from patients with STEMI remains unclear. This prompted us to explore the circulating levels of IL-17 and its effect on the recruitment and activation of monocyte subsets derived from STEMI and post-STEMI patients.

## 2. Materials and Methods

### 2.1. Experimental Protocol

The study was approved by the Human Ethics and Medical Research Committee of the Instituto Mexicano del Seguro Social (IMSS) on April 30, 2013, and registered (R-2013-785-030). It was conducted according to the Helsinki Declaration guidelines, and all patients provided written informed consent.

### 2.2. Patient Population

This study included 65 patients evaluated during STEMI (patients who had an acute myocardial infarction with ST-segment elevation and successfully treated with primary angioplasty within the first 24 hours) and post-STEMI (patients who had an acute myocardial infarction with ST-segment elevation and successfully treated with primary angioplasty five days after the onset of STEMI) who were admitted to the Hospital de Cardiología, Centro Médico Nacional Siglo “XXI”, IMSS. The plasma levels of cytokines in the 65 patients were determined, and 11 of these patients were included in the experimental assay. STEMI was diagnosed with the following criteria: (1) chest pain > 30 minutes, with or without shortness of breath, sweating, nausea, and/or vomiting; (2) ST-segment elevation and/or abnormal Q-wave on an electrocardiogram and/or the presence of an emerging left block bundle branch; and (3) an elevated troponin level, specifically 10% above the 99th percentile of the upper limit of the reference value, or an elevated creatinine kinase MB isoenzyme (CK MB) level, higher than the 99th percentile of the upper limit of the reference value. The exclusion criteria included the following: (1) hemodynamic instability or electrical shock; (2) mechanical complications of infarction; (3) presence of malignancies, hematological or immunological disorders, or any other inflammatory condition or infection likely to be associated with the acute phase response; (4) previous immunosuppressive or anti-inflammatory therapy; and (5) a serum creatinine level ≥ 1.5 mg/dl or known allergy to iodine contrast medium.

### 2.3. Plasma Cytokine Determinations

Plasma samples were obtained from the 65 STEMI and post-STEMI patients. The plasma levels of CX3CL1, CCL2, and IL-17A were analyzed using a ProcartaPlex multiplex assay according to the manufacturer's instructions (Merck Millipore, Darmstadt, Germany). The detection limit was >0.5 pg/ml for CX3CL1, 37.5 pg/ml for CCL2, and 2.4 pg/ml for IL-17A.

### 2.4. Human Umbilical Vein Endothelial Cells

Human umbilical vein endothelial cells (HUVECs) were obtained from human umbilical cords [15], and the cells were cultured at 37°C in a humidified atmosphere with 5% CO_2_ using an Endothelial Cell Growth Medium (EGM-2) medium bullet kit (Lonza, Verviers, Belgium). Cells in their third or fourth passage were used for all the reported experiments. Recuperated cells were stained with anti-CD31 FITC-conjugated, anti-CD309 (KDR/VEGFR2) APC-conjugated, and anti-CD146 PE-conjugated antibodies (BD Biosciences, San Jose, California, USA) and evaluated with a MACS Quant Analyzer 10 cytometer (Miltenyi Biotec, Bergisch Gladbach, Germany) flow cytometer; molecular analysis was performed with FlowJo software version 7.6.5 (TreeStar, Inc.).

### 2.5. Monocyte Isolation

Peripheral blood mononuclear cells (PBMCs) were obtained from patients on days 1 (STEMI) and 5 (post-STEMI) after the onset of STEMI by density centrifugation using Lymphoprep (Axis-Shield, Oslo, Norway). Blood samples were mixed with an equal volume of phosphate-buffered saline (PBS), pH 7.4, layered over 3 ml of Lymphoprep, and centrifuged at 700 × g for 30 minutes. The recovered PBMCs were washed three times with PBS (pH 7.4). Classical monocytes (CD14^+^CD16^−^) were then isolated from the PBMCs using Monocyte Isolation Kit II (Miltenyi Biotec, Bergisch Gladbach, Germany), and intermediate/nonclassical monocytes were isolated in two phases. In the first phase, all monocytes were enriched using human Pan Monocyte Isolation Kit (Miltenyi Biotec); in the second phase, intermediate/nonclassical monocytes were isolated using magnetic microbeads coupled to an anti-CD16 monoclonal antibody. The purified cells were stained for CD14; the purity of the classical monocytes was >93%, and that of the intermediate/nonclassical monocytes was >89%.

### 2.6. Transendothelial Migration Assay

HUVECs were grown overnight on 1% gelatin-coated porous membranes in a Transwell chamber (Corning Inc., Cambridge, USA) of 6.5 mm diameter, and 5 *μ*m pore size until a monolayer was formed. The HUVECs were activated for 1 day with IL-17 (60 ng/ml), interferon- (IFN-) *γ* (25 ng/ml), which was used as an inhibitory control for monocyte migration, or IL-17/IFN-*γ* (R&D Systems, Minnesota, USA), and culture medium alone was used as a negative control. A total of 3 × 10^5^ monocytes in 50 *μ*l medium were added to the upper chamber. In the transendothelial migration assay, serum-free RPMI medium was used in both compartments, and culture medium alone was used as a negative control. CCL2 (10 ng/ml) and CX3CL1 (25 ng/ml) were used as positive controls for classical monocytes and intermediate/nonclassical monocytes, respectively. After 3 hours, the migrating monocytes were recovered and quantitated by flow cytometry. The concentrations of CCL2 and CX3CL1 used were obtained through curves (Additional files).

### 2.7. Expression of Receptors

A total of 3 × 10^5^ CD14^+^CD16^−^ and CD14^++^CD16^+^/CD14^+^CD16^++^ monocytes were cultured for 1 day with IL-17 (60 ng/ml), IFN-*γ* (25 ng/ml), which was used as a positive control of monocyte activation, or IL-17/IFN-*γ* (R&D Systems); culture medium alone was used as a negative control. The monocyte subsets were then stained with anti-TLR (Toll-like receptor) 4 PE-conjugated, anti-CD86 PECy5-conjugated, and anti-HLA-DR FITC-conjugated antibodies (BioLegend, San Diego, CA, USA) or isotype control antibodies for 20 minutes in the dark at 4°C. The cells were washed twice with PBS containing 1% BSA and 1% sodium azide. The expression levels of different molecules were measured using a MACS Quant flow cytometer (Miltenyi Biotec), and the mean fluorescence intensity (MFI) of each sample was quantified using FlowJo software (TreeStar, Inc.). The concentrations of IL-17 and IFN-*γ* used were obtained through curves (Additional files).

### 2.8. TNF-*α* and IL-6 Assays

TNF-*α* and IL-6 levels in supernatant were measured using an enzyme-linked immunosorbent assay (ELISA) (eBioscience, San Diego, USA) according to the manufacturer's instructions. The measurements in each assay were performed in duplicate. The detection limit was 1.65 pg/ml for TNF-*α* and <2 pg/ml for IL-6.

### 2.9. Statistical Analysis

All statistical analyses were performed using GraphPad Prism version 7 (GraphPad Software, Inc., San Diego, CA, USA), and a level of *p* < 0.05 was considered statistically significant. Levels of CCL2, CX3CL1, and IL-17, which are presented as medians (interquartile ranges), were analyzed with the Wilcoxon test. Multiple comparisons of the groups were analyzed by the Mann-Whitney *U* and Kruskal-Wallis tests (data shown in the figures are expressed as the mean ± SEM). The experimental study included eleven independent experiments and measured circulating cytokine levels in sixty-five patients.

## 3. Results

### 3.1. Patient Characteristics

The characteristics of the study population are shown in Table 1. The STEMI patients included 51 men and 14 women, with a mean age of 63 ± 10 years (range, 37 to 83 years). Of these patients, 31 had diabetes, 32 had systemic arterial hypertension, 42 were smokers, 22 had obesity, and 30 had hyperlipidemia. The mean value of the maximum creatine kinase (CK) level was 2.216 ± 1.850 IU/l, the maximum CK-MB level was 196 ± 188.1, and the maximum creatine phosphokinase (CPK) level was 2.730 ± 1.827.

### 3.2. Cytokine Levels in Patients with STEMI and Post-STEMI

**Figure 1:**
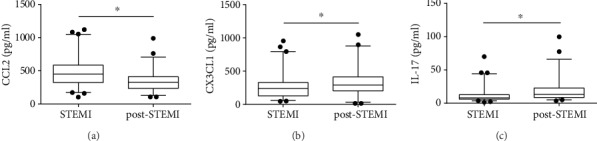
Circulating cytokines during STEMI and post-STEMI. Patient plasma was obtained during STEMI and post-STEMI, and the circulating levels of (a) CCL2, (b) CX3CL1, and (c) IL-17 were assessed. The concentrations of the cytokines in the plasma were determined by a multiplex assay. *n* = 65.

Cytokine secretion is particularly active after myocardial infarction and contributes to cellular recruitment and activation cellular [2, 16, 17]. To obtain a picture of the circulating levels of chemokines that contribute to the recruitment of monocytes and IL-17 in patients, we determined CCL2, CX3CL1, and IL-17 in the plasma of STEMI and post-STEMI patients. Plasma CCL2 levels were higher in patients with STEMI and decreased post-STEMI (Figure 1(a)) (CCL2: 497.2 ± 229.3 pg/ml vs. 346.3 ± 162.6 pg/ml, *p* = 0.0001). Conversely, circulating plasma CX3CL1 levels were lower in STEMI patients than in post-STEMI patients (CX3CL1: 271.0 ± 192.9 pg/ml vs. 334.8 ± 224.8 pg/ml, *p* = 0.0094), as shown in Figure 1(b). These findings suggest that CCL2 and CX3CL1 might facilitate the mobilization of circulating CD14^++^CD16^−^ and CD14^++^CD16^+^/CD14^+^CD16^++^ monocytes, respectively. Interestingly, IL-17 levels were higher in post-STEMI patients than in STEMI patients, as shown in Figure 1(c) (IL-17: 12.25 ± 11.69 vs. 19.09 ± 17.86, *p* = 0.0001). This result suggests that IL-17 may favor proinflammatory responses to induce cell activation after AMI.

### 3.3. Effect of IL-17 on the Transendothelial Migration of Monocyte Subsets

**Figure 2:**
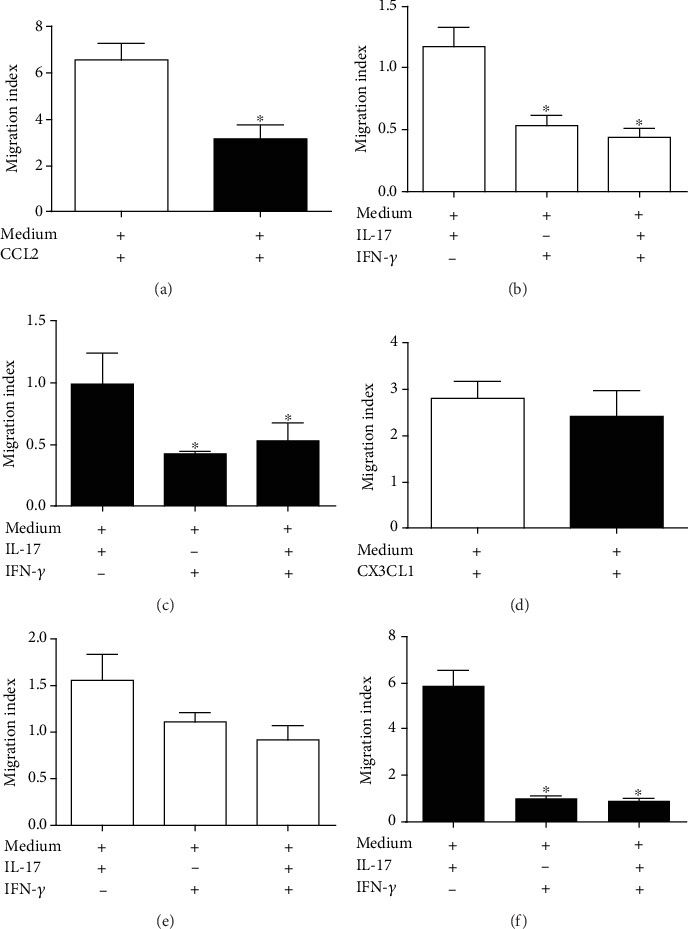
Monocytes transmigrate across a human umbilical vein endothelial cell monolayer in response to IL-17. A sample of 3 × 10^5^ (a–c) CD14^++^CD16^−^ or (d–f) CD14^++^CD16^+^/CD14^+^CD16^++^ monocytes was added to the upper surface of monolayers of human umbilical vein endothelial cells (HUVECs) previously treated with IL-17, IFN-*γ*, or IL-17/IFN-*γ*; culture medium alone was used as a negative control. The monocytes were allowed to transmigrate across the HUVEC monolayers in the presence of CCL2, CX3CL1, or culture medium for 3 hours. All data are presented as the migration index, which relates the number of cells that migrated in response to the indicated stimulus to the number of cells that migrated in response to the negative control. White column: STEMI: black column: post-STEMI; *n* = 11. ^∗^*p* < 0.05.

The recruitment of monocyte subsets toward damaged tissue requires migration of monocytes through the endothelium [16–18]. First, we examined the transmigration of monocyte subsets from STEMI and post-STEMI patients in response to CCL2 and CX3CL1 and found that CD14*^+^*CD16*^−^* monocytes from the former patients migrated more than those derived from the latter patients in response to CCL2 (Figure 2(a)). Moreover, CX3CL1 induced, in a similar way, the transmigration of CD14^++^CD16^+^/CD14^+^CD16^++^ monocytes in patients during STEMI and post-STEMI (Figure 2(d)). IL-17 has the ability to promote inflammation through the induction of cytokines and chemokines, which contribute to the migration of monocytes [8, 19, 20]. To characterize the role of IL-17 in the migration of monocyte subsets through the endothelium in patients with STEMI, we assessed whether treatment of HUVECs with IL-17 results in an increased migration of monocyte subsets across the endothelial monolayer. Indeed, treatment of HUVECs with IL-17 induced the transmigration of CD14^++^CD16^−^ monocytes from patients during STEMI and post-STEMI (Figures 2(b) and 2(c)). However, treatment of HUVECs with IFN-*γ* (inhibition of migration control for monocytes) and the combination of IL-17/IFN-*γ* resulted in lower migration of CD14^++^CD16^−^ monocytes than with IL-17 alone. Nonetheless, IL-17 induced the migration of CD14^++^CD16^+^/CD14^+^CD16^++^ monocytes from STEMI patients via HUVECs, and IL-17 increased the migration of CD14^++^CD16^+^/CD14^+^CD16^++^ monocytes from post-STEMI patients (Figures 2(e) and 2(f)) in relation to IFN-*γ* or IL-17/IFN-*γ*. These results suggest that IL-17 similarly contributes to the transmigration of CD14*^+^*CD16*^−^* monocytes in STEMI and post-STEMI and considerately enhances the migration of CD14^++^CD16^+^/CD14^+^CD16^++^ monocytes in post-STEMI versus STEMI.

### 3.4. Effect of IL-17 on Markers in Monocyte Subsets

**Figure 3:**
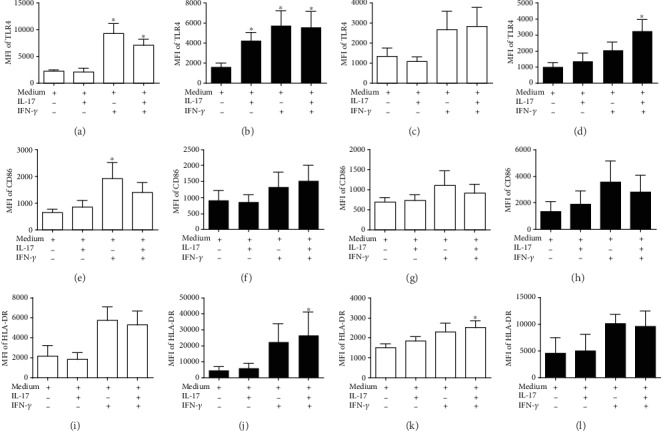
Expression of surface markers on monocytes stimulated with IL-17. Human CD14^++^CD16^−^ and CD14^++^CD16^+^/CD14^+^CD16^++^ monocytes were treated with IL-17, IFN-*γ*, which was used as a positive control, IL-17/IFN-*γ*, or culture medium alone, which was used as a negative control, for 24 hours. Human CD14^++^CD16^−^ in STEMI: (a) TLR4, (e) CD86, and (i) HLA-DR. Human CD14^++^CD16^−^in post-STEMI: (b) TLR4, (f) CD86, and (j) HLA-DR. Human CD14^++^CD16^+^/CD14^+^CD16^++^ monocytes in STEMI: (c) TLR4, (g) CD86, and (k) HLA-DR. CD14^++^CD16^+^/CD14^+^CD16^++^ monocytes post-STEMI: (d) TLR4, (h) CD86, and (l) HLA-DR. Expression levels of TLR4, CD86, and HLA-DR are expressed as MFI. White column: STEMI; black column: post-STEMI; *n* = 11. ^∗^*p* < 0.05.

IL-17 is an inflammatory cytokine that induces the activation of myeloid lineage cells [12]. In this context, we treated the two patient monocyte subsets (obtained during STEMI or post-STEMI) with IL-17 and evaluated the markers TLR4, CD86, and HLA-DR. Stimulation of CD14*^+^*CD16*^−^* or CD14^++^CD16^+^/CD14^+^CD16^++^ monocytes from patients with STEMI with IL-17 did not affect expression of TLR4 (Figures 3(a) and 3(c)). However, IL-17 treatment of CD14*^+^*CD16*^−^* and CD14^++^CD16^+^/CD14^+^CD16^++^ monocytes from patients post-STEMI resulted in a 2.6-fold and 1.3-fold increase in the expression of TLR4, respectively, compared to unstimulated cells (Figures 3(b) and 3(d)). In contrast, IL-17 did not affect expression of CD86 (Figures 3(e)–3(h)) or HLA-DR (Figures 3(i)–3(l)) on CD14*^+^*CD16*^−^* and CD14^++^CD16^+^/CD14^+^CD16^++^ from STEMI and post-STEMI patients. Additionally, IFN-*γ* (positive control) and IL-17/IFN-*γ* treatment significantly increased the levels of TLR4, CD86, and HLA-DR in STEMI and post-STEMI monocyte subsets; CD14^++^CD16^+^/CD14^+^CD16^++^ monocytes treated with IL-17/IFN-*γ* exhibited a 1.6-fold increase in expression of TLR4 compared with monocytes treated with IFN-*γ*. These results suggest that IL-17 slightly affects expression of molecules related to pattern recognition receptors such as TLR4 in both monocyte subsets in post-STEMI patients, which may contribute to the inflammatory response.

### 3.5. Effect of IL-17 on the Secretion of Proinflammatory Cytokines in Monocyte Subsets

**Figure 4:**
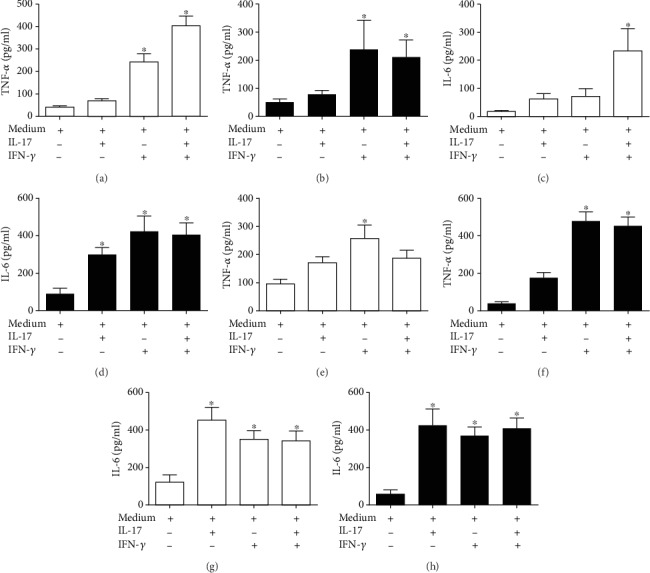
IL-17 induces the secretion of proinflammatory cytokines in monocyte subsets. Human CD14^++^CD16^−^ or CD14^++^CD16^+^/CD14^+^CD16^++^ monocytes were treated with IL-17, IFN-*γ*, which was used as a positive control, IL-17/IFN-*γ*, or culture medium alone, which was used as a negative control, for 24 hours. Human CD14^++^CD16^−^in STEMI: (a) TNF-*α* and (c) IL-6. Human CD14^++^CD16^−^in post-STEMI: (b) TNF-*α* and (d) IL-6. Human CD14^++^CD16^+^/CD14^+^CD16^++^ monocytes in STEMI: (e) TNF-*α* and (g) IL-6. CD14^++^CD16^+^/CD14^+^CD16^++^ monocytes post-STEMI: (f) TNF-*α* and (h) IL-6. Concentrations of TNF-*α* and IL-6 in the culture supernatants were determined by ELISA. White column: STEMI; black column: post-STEMI; *n* = 11. ^∗^*p* < 0.05.

Next, we determined the effect of IL-17 on CD14*^+^*CD16*^−^* and CD14^++^CD16^+^/CD14^+^CD16^++^ monocytes with regard to the secretion of cytokines. Monocytes are essential cells in the inflammatory response during infarction [2, 7, 4]. CD14*^+^*CD16*^−^* monocytes from STEMI and post-STEMI patients treated with IL-17 exhibited increased the levels of TNF-*α* (Figures 4(a) and 4(b)). However, CD14^++^CD16^+^/CD14^+^CD16^++^ monocytes from post-STEMI patients cultured with IL-17 displayed 2.6-fold increased production of TNF-*α* compared with cells cultured with medium alone (Figures 4(e) and 4(f)). IL-17 induced a 3.4-fold increase in IL-6 levels in CD14*^+^*CD16*^−^* monocytes from STEMI patients and a 1.9-fold increase in post-STEMI patients compared with monocytes cultured in medium alone (Figures 4(c) and 4(d)). In addition, IL-17 treatment of CD14^++^CD16^+^/CD14^+^CD16^++^ monocytes from STEMI and post-STEMI patients caused 3.7-fold and 7.4-fold increases in IL-6, respectively, in relation to monocytes cultured only with medium alone (Figures 4(g) and 4(h)). We also found that IFN-*γ* and the combination of IL-17/IFN-*γ* increased TNF-*α* and IL-6 levels in CD14*^+^*CD16*^−^* and CD14^++^CD16^+^/CD14^+^CD16^++^ monocytes from patients with STEMI and post-STEMI. These results suggest that IL-17 induces the activation of CD14*^+^*CD16*^−^* and CD14^++^CD16^+^/CD14^+^CD16^++^ monocytes to produce inflammatory cytokines, which might contribute to the inflammatory response in STEMI and post-STEMI.

## 4. Discussion

This study demonstrates for the first time higher circulating levels of IL-17 post-STEMI than during STEMI. Additionally, IL-17 differentially induces the migration and activation of CD14^+^CD16^−^ and CD14^++^CD16^+^/CD14^+^CD16^++^ monocytes during STEMI and post-STEMI.

During AMI, cytokines in the circulation play essential roles in the recruitment of cells to damaged tissue and in the activation of cells of the innate immune system [2]. We found that patients with STEMI had high levels of CCL2 and that these levels decreased post-STEMI [21–24], which is crucial for CD14^++^CD16^−^ monocyte recruitment [16, 17] and monocyte infiltration into the infarcted area [25]. On the other hand, we found higher levels of CX3CL1 in post-STEMI patients than in STEMI patients. Previous reports have shown increased circulating levels of CX3CL1 in patients with STEMI [26]. Additionally, *in vivo* studies in mouse models of AMI found an increase in the expression of CX3CL1 during phase 2 of infarction, which suggested the mobilization of Ly-6C^low^ monocytes (mouse counterparts of human CD14^++^CD16^+^/CD14^+^CD16^++^ monocytes) [4, 6]. Our results also demonstrate that patients had higher levels of IL-17 in the circulation post-STEMI than during STEMI. Several reports have shown elevated levels of IL-17 in infarction and even a significant increase in IL-17 expression have been observed during the first hours of STEMI [13, 27]. These results suggest that IL-17 might have proinflammatory activity in STEMI and post-STEMI.

In this context, we hypothesize that circulating IL-17 in STEMI and post-STEMI patients might be essential for endothelial activation and the subsequent recruitment and activation of CD14^++^CD16^−^ and CD14^++^CD16^+^/CD14^+^CD16^++^ monocytes, which may induce the secretion of proinflammatory cytokines. IL-17 is a cytokine whose level increases after AMI [28]. These findings suggest that IL-17 may be involved in the pathophysiology of infarction. We demonstrate that IL-17 contributes to similar transmigration of CD14^+^CD16*^−^* monocytes from STEMI and post-STEMI patients through a HUVEC monolayer; we also found that IL-17 preferentially contributes to the transmigration of CD14^+^CD16^+^/CD14^+^CD16^++^ monocytes from post-STEMI patients. In accordance with our results, *in vitro* studies have shown that IL-17 contributes to the recruitment of mononuclear cells, such as monocytes, through its ability to induce expression of CCL2 and increase that of adhesion molecules, such as selectin E, ICAM-1, and VCAM-1, in HUVECs [11, 13, 29]. Moreover, direct inhibition of IL-17 in Apoe^−/−^ mice causes a considerable reduction in the accumulation of macrophages in atherosclerotic plaques [30]. Additionally, IFN-*γ* and IL-17/IFN-*γ* reduced the migration of CD14^+^CD16*^−^*and CD14^+^CD16^+^/CD14^+^CD16^++^ monocytes through a HUVEC monolayer, which is consistent with previous reports [31, 32]. On the other hand, we found only slight increases in TLR4, CD86, and HLA-DR expression on CD14^+^CD16*^−^* and CD14^+^CD16^+^/CD14^+^CD16^++^ monocytes in response to IL-17, and this effect was most evident for TLR4, suggesting an increase in the response to danger-associated molecular patterns post-STEMI. Previous reports have indicated that IL-17 in human macrophages induces the expression of TLR4, which is essential in the inflammatory response [2, 33]. Additionally, we show that IFN-*γ* or the combination of IL-17 and IFN-*γ* markedly increased the expression levels of TLR4, CD86, and HLA-DR on monocytes from STEMI and post-STEMI patients. IFN-*γ* plays an essential role in the inflammatory response through activation of monocytes [34] and shows a synergistic effect with IL-17 [35].

Monocytes that infiltrate into ischemic cardiac tissue can be activated by inflammatory mediators such as IL-17 [2, 20]. According to our results, CD14^++^CD16^−^ and CD14^++^CD16^+^/CD14^+^CD16^++^ monocytes produce low levels of TNF-*α* in response to IL-17, both during STEMI and post-STEMI. However, post-STEMI CD14^++^CD16^+^/CD14^+^CD16^++^ monocytes exhibited increased secretion of TNF-*α* after stimulation with IL-17. Additionally, we showed that IL-17 induced a slight increase in IL-6 production in CD14^+^CD16 monocytes derived from patients during STEMI, and this level of IL-6 was markedly increased in monocytes from patients post-STEMI. IL-17 was also able to enhance secretion of IL-6 by CD14^++^CD16^+^/CD14^+^CD16^++^ monocytes from STEMI patients and lower secretion by the corresponding post-STEMI monocytes. Previous reports have found that IL-17 induces secretion of TNF-*α* and IL-6 by human monocytes and macrophages [12, 36]. Similarly, blocking IL-17 decreases TNF-*α* and, to a larger magnitude, IL-6 production in atherosclerotic lesions in mouse models [29]. Our results suggest that IL-17 acts on CD14^++^CD16^−^ monocytes in the post-STEMI environment and on CD14^++^CD16^+^/CD14^+^CD16^++^ monocytes in both the STEMI and post-STEMI environments, indicating that IL-17 induces a proinflammatory effect on both monocyte subsets. This likely occurs through IL-6 production, which may contribute to inflammation in cardiac tissue during both stages of infarction. Additionally, our results demonstrate that IFN-*γ* or the combination of IL-17/IFN-*γ* induced a proinflammatory effect on both monocyte subsets during STEMI and post-STEMI, though we did not find an additive or synergistic effect with the combination of IFN- *γ* and IL-17.

## 5. Conclusions

In conclusion, we show a gradual increase in IL-17 in STEMI and post-STEMI patients. IL-17 increases the expression of TLR4 on CD14*^+^*CD16*^−^* and CD14^++^CD16^+^/CD14^+^CD16^++^ cells post-STEMI. Moreover, IL-17 has a potential role in the recruitment of CD14^++^CD16^+^/CD14^+^CD16^++^ monocytes post-STEMI. In addition, IL-17 contributes to secretion of IL-6 by CD14^++^CD16^−^ and CD14^++^CD16^+^/CD14^+^CD16^++^ monocytes in STEMI and post-STEMI. These findings for an *in vitro* model suggest that in STEMI and post-STEMI patients, IL-17 induces the recruitment and activation of monocyte subsets through an increase in TLR4 and IL-6 secretion, which might cause damage to myocardial tissue in STEMI and post-STEMI.

## Figures and Tables

**Table 1:** Characteristics of the population on day 1.

Patient characteristics	
Characteristic	STEMI (*n* = 65)
Age (years)	70 (63-80)
Sex, male/female	51/14
Diabetes mellitus, *n* (%)	31 (47)
Hypertension, *n* (%)	32 (49)
Hyperlipidemia, *n* (%)	30 (46)
Smoking, *n* (%)	42 (64)
Obesity, *n* (%)	22 (33)
Statin, *n* (%)	63 (96)
Beta-blocker, *n* (%)	31 (47)
ACE/ARB, *n* (%)	34 (52)
Aspirin, *n* (%)	63 (96)
Serum creatinine (mg/dl)	1.0 (0.9-1.3)
Max CK (IU/l)	2216 (1361-3438)
Max CK-MB (IU/l)	196 (117-340)
Glucose (mg/dl)	130 (130-218)
Total cholesterol (mg/dl)	138 (125-176)
Triglyceride (mg/dl)	140 (106-175)
Body mass index (kg/m^2^)	26.7 (24.2-29.4)

## Data Availability

Data are available upon request and may be obtained by contacting the corresponding author.

## Abbreviations

STEMI: ST-segment elevation myocardial infarction
TNF: Tumor necrosis factor
IL: Interleukin
AMI: Acute myocardial infarction
ICAM: Intercellular adhesion molecules
Apoe: Apolipoprotein E
CK MB: Creatine kinase MB isoenzyme
HUVECs: Human umbilical vein endothelial cells
PBMCs: Peripheral blood mononuclear cells
PBS: Phosphate-buffered saline
IFN: Interferon
TLR: Toll-like receptor
MFI: Mean fluorescence intensity
CK: Creatine kinase
CPK: Creatine phosphokinase
ELISA: Enzyme-linked immunosorbent assay.

## Supplementary Materials

Supplementary Materials Figure S1: effect of CCL2, CX3L1, and IFN-*γ* on migration of monocytes. Figure S2: assay of IL-17 and IFN-*γ* on monocytes.
