# Supplementary material for: Effect of Interleukin-17 in the Activation of Monocyte Subsets in Patients with ST-Segment Elevation Myocardial Infarction
Source: J Immunol Res. 2020 Jun 27;2020:5692829. doi: 10.1155/2020/5692829 (PMC7336211; doi:10.1155/2020/5692829)
Supplement: Supplementary Materials — Figure S1: effect of CCL2, CX3L1, and IFN-γ on migration of monocytes. Figure S2: assay of IL-17 and IFN-γ on monocytes. [file 5692829.f1.pdf]

**Supplementary Materials**

**Figure S1. Effect of CCL2, CX3L1 and IFN-γ on migration of monocytes.** HUVECs were grown overnight on 1% gelatin-coated porous membranes in a Transwell chamber (Corning Inc, Cambridge, Massachusetts, USA) with a 6.5-mm diameter and 5µm-pore size. In the lower chamber were added individually concentrations of CCL2 (5, 7.5 and 10 ng/ml), CX3CL1 (25, 50 and 100 ng/ml) and IFN-γ (25, 50 and 100 ng/ml), culture medium alone was used as a negative control. In the assay of IFN-γ was used CCL2 as a positive control. Peripheral blood mononuclear cells were obtained from buffy coasts and total monocytes 3×105 (obtained with Pan Monocyte Isolation Kit from Miltenyi Biotec, Bergisch Gladbach, German) in 50 µl medium were added to the upper chamber. After 3 h, the migrating monocytes were recovered and quantitated by flow cytometry, n=6. * $p < 0.05$.

**Figure S2. Assay of IL-17 and IFN-γ on monocytes**

Peripheral blood mononuclear cells were obtained from buffy coasts and total monocytes 3×105 (obtained with Pan Monocyte Isolation Kit from Miltenyi Biotec, Bergisch Gladbach, German) were treated with IL-17 (15, 30 and 60 ng/ml) for 24 hours at 37ºC. Alternatively, total monocytes were treated with 15, 20 and 25 ng/ml IFN□ for 24 hours at 37ºC. As a negative control the monocytes were cultured only with culture medium. The concentrations of IL-6 in the culture supernatants of monocytes were determined by ELISA, according to the manufacturer's instructions, n=6. *$p < 0.05$.

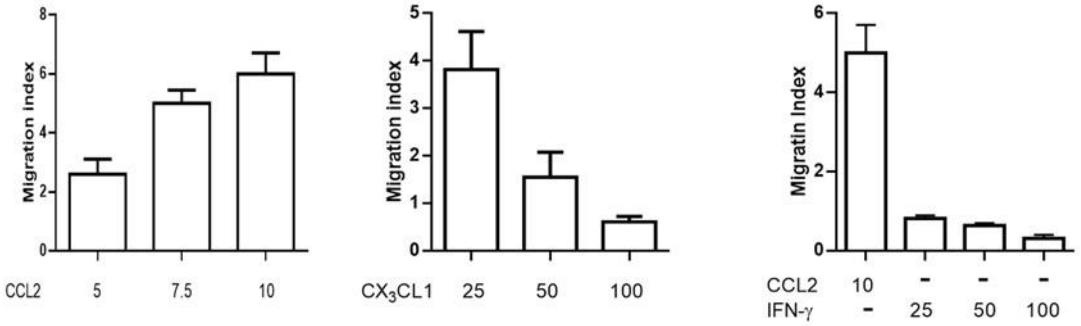


**Figure S1**

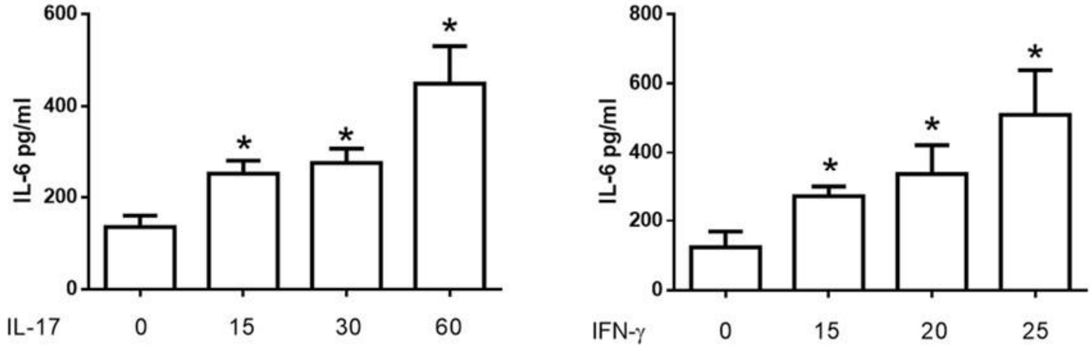


**Figure S2**
